# Cerebellar Syndrome From Proton Pump Inhibitor–Induced Hypomagnesemia: Two Reversible but Relapsing Cases

**DOI:** 10.1155/crot/8815667

**Published:** 2025-12-08

**Authors:** Valentine Léonard, Brieuc Gevers, Jean-Philippe Van Damme, Pierre Garin, Michaël Hardy

**Affiliations:** ^1^ Otorhinolaryngology Department, CHU UCL Namur, UCLouvain, Yvoir, Belgium; ^2^ Anesthesiology Department, CHU UCL Namur, UCLouvain, Yvoir, Belgium

**Keywords:** cerebellar syndrome, hypomagnesemia, proton pump inhibitors, vertigo

## Abstract

**Introduction:**

Cerebellar syndromes are rare yet potentially serious presentations in the emergency department, requiring timely diagnosis and intervention. The differential diagnosis is wide, encompassing stroke, multiple sclerosis, toxic reactions, and metabolic imbalances such as hypomagnesemia. Proton pump inhibitors (PPIs) have been increasingly recognized as a cause of severe hypomagnesemia, which can lead to reversible cerebellar syndrome.

**Case Reports:**

We present two cases of reversible cerebellar syndrome caused by hypomagnesemia. The first case involves a 65‐year‐old man with a two‐week history of weakness, dizziness, and inappetence. After extensive investigation, including normal MRI and vestibular testing, his symptoms were attributed to severe hypomagnesemia secondary to esomeprazole use. Magnesium supplementation led to clinical improvement. The second case is a 68‐year‐old man with progressively worsening dizziness, nausea, and instability. Hypomagnesemia related to omeprazole use was identified as the cause, and intravenous magnesium supplementation significantly improved his condition.

**Discussion:**

Hypomagnesemia is an often overlooked but important cause of cerebellar dysfunction. Magnesium deficiency leads to neuronal hyperexcitability, particularly affecting the cerebellum. In both cases, PPI use was the primary cause of hypomagnesemia, with magnesium supplementation reversing most symptoms. Cerebellar symptoms from hypomagnesemia may fluctuate, complicating diagnosis, and normal brain imaging does not rule out this condition. Regular monitoring of serum magnesium levels in patients on long‐term PPI therapy is essential. These cases illustrate that PPI‐induced hypomagnesemia should be considered in patients presenting with atypical dizziness and balance disorders. Early diagnosis and treatment with magnesium supplementation can reverse symptoms, although some residual effects may persist. Continuous monitoring is crucial for patients requiring long‐term PPI therapy to prevent recurrence.

## 1. Introduction

Cerebellar syndrome is a rare clinical presentation in the emergency department, but it represents a diagnostic emergency, as it may indicate an acute pathology requiring prompt management. The clinical presentation is characterized by gait and balance and coordination disorders with ataxia, dysarthria, vertigo, and nystagmus [1]. The differential diagnosis is broad and includes acute vascular problems (stroke); multiple sclerosis; paraneoplastic syndromes; various toxic or drug reactions; and metabolic, infectious, or inflammatory problems (e.g., Guillain–Barré syndrome). Among these, a rare and misleading cause is hypomagnesemia [1].

Magnesium is the second most frequent ion in the intracellular compartment (after potassium) and the fourth most frequent ion in the extracellular compartment (after calcium, potassium, and sodium) [2]. Normal blood concentration is 0.65–1.05 mmol/L (1.30–2.10 mEq/L). It plays a crucial role in various physiological processes, including neurological function. It is involved in nerve transmission, cardiac excitability, neuromuscular conduction, muscular contraction and relaxation, vascular tone, and glucose and insulin metabolism [2]. It is a cofactor for numerous enzymatic reactions. Magnesium homeostasis is based on dietary intake and intestinal absorption, stool excretion, uptake and release from bones, and balance between intra‐ and extracellular compartments.

Hypomagnesemia is not uncommon, reported in 2.5%–15% of the general population, even more in hospitalized and intensive care unit patients [3]. It may occur in cases of insufficient food intake, increased intestinal or renal losses, or inadequate redistribution from the extracellular to the intracellular compartment [3]. Some drugs are also known to cause hypomagnesemia, such as proton pump inhibitors (PPIs), diuretics, antibiotics, antivirals, antimycotics, chemotherapy, immunosuppressants, and cardiac medications.

In this manuscript, we present two cases of atypical hypomagnesemia‐induced reversible cerebellar syndrome (HiCS) attributed to PPIs.

## 2. Case 1

A 65‐year‐old man presented to the emergency room with a two‐week history of generalized weakness and loss of appetite.

His medical history included a right superior lobectomy, 1 month before, for a pulmonary adenocarcinoma, an epidermoid carcinoma of the left vocal cord treated by cordectomy, a coronary angioplasty, arterial hypertension, post‐tabagic COPD Gold 1, restless legs syndrome, Type II diabetes, ethylism, and active smoking.

His medication included aspirin 80 qd, esomeprazole 40 mg, diltiazem 200 mg qd, lormetazepam 1 mg qd, metformin 425 mg bd, perindopril 10 mg qd, atorvastatin 40 mg qd, ezetimibe 10 mg qd, and inhaled tiotropium 18 μg qd.

The patient complained of loss of appetite, generalized weakness, dizziness with postural instability, blurred vision, and nausea and vomiting for a few days. No abdominal or thoracic pain or fever was reported. The blood pressure was 150/93 mmHg, temperature was 36,2°C, cardiac frequency was 91 beats per minute, and the oxygen level was 99%.

The general clinical examination was unremarkable. The neurological evaluation identified a multidirectional nystagmus without alteration of the state of consciousness, abnormality of the cranial pairs, or sensory‐motor deficit.

A blood test showed an inflammatory syndrome with a CRP level at 80 mg/L, leucocytes at 13,93/μL, glycemia at 182 mg/dL, urea levels at 34 mg/dL, creatine levels at 0.46 mg/dL, sodium levels at 138 mmol/L, and potassium levels at 3.6 mmol/L.

He also had a normal electrocardiogram, an encysted effusion in the right upper pulmonary apex on a chest X‐ray, and a normal head CT‐scan.

The diagnosis of a pulmonary infection was initially made, and antibiotics were started on the recommendation of the pneumologist. The attending neurologist confirmed significant gait instability, normal cerebellar tests, and a slight dysphonia, which was related to the vocal cord surgery. The neurological examination was otherwise unremarkable. The Hallpike maneuver triggered a leftward‐beating nystagmus. An ENT consultation and an MRI were requested.

The ENT evaluation revealed spontaneous multidirectional nystagmus, retropulsion with the Romberg, left dysmetria with the finger–nose maneuver, and a normal head impulse test. A central etiology was suspected, and an MRI was carried out quickly. The brain MRI and lumbar puncture were normal, and the patient was discharged home with an ENT check‐up scheduled later.

After 10 days, the patient was readmitted to the emergency department with dizziness on movement and at rest, with severe nausea leading to total inappetence. The patient was admitted to the ENT department. Vestibular examinations were performed. The vertical test was not deviated. Videonystagmography (VNG) revealed no spontaneous nystagmus, no nystagmic preponderance in the damped pendulum test, and no hyporeflexia in the caloric tests. Ocular fixation indices were pathological (87%). Visual pursuit and saccades were correctly performed. The video head impulse test (VHIT) showed no clear semicircular canal involvement.

The cerebellar syndrome was finally attributed to severe hypomagnesemia (measured < 0.24 mmol/L 4 days after hospital admission; local reference range: 0.66–0.94 mmol/L), and an oral supplementation was administered. As there was no urinary loss (normal magnesuria) and no signs of gastrointestinal disease, the hypomagnesemia was attributed to PPI treatment. The dose of PPI was gradually reduced from 40 to 20 mg qd, but as the patient’s reflux could only be relieved by PPI, it was decided to keep the PPI while maintaining the magnesium supplementation.

The patient was readmitted to the hospital with recurrence of dizziness and instability 5 months later. He reduced the magnesium dosage to 450 mg once a day, with a blood magnesium concentration again below the limit of quantification (0.24 mmol/L). The twice‐a‐day dosage was restored, which enabled magnesium levels to be maintained slightly below local reference values.

## 3. Case 2

A 68‐year‐old man presented to the emergency department with dizziness and vomiting that had been progressively worsening for 1 month. Patient’s history included arterial hypertension, arteritis of the lower limbs, hypertensive hypertrophic cardiomyopathy, postsmoking COPD Gold 2, gastroesophageal reflux disease, paroxysmal atrial fibrillation, and active smoking. His medication included omeprazole 20 mg qd, asaflow 80 mg qd, apixaban 5 mg bid, bisoprolol 2.5 mg qd, simvastatin 40 mg qd, lisinopril 5 mg qd, and alprazolam 0.25 mg qd.

The patient complained of baseline permanent vertigo (whether stationary or moving) with intermittent but intense acute vertigo lasting a few seconds or minutes, with no identified trigger. He also complained of nausea for 1 month, progressively worsening, associated with inappetence and an altered general state. He was now experiencing constant dizziness with permanent instability and repeated vomiting for 3 days. Walking was currently impossible because of the dizziness, and he could no longer stand on his legs.

General physical examination and basic neurological examination in the emergency room were unremarkable. The finger–nose test was slow but with no obvious asymmetry. The Romberg and Fukuda tests were not possible due to severe dizziness.

The blank brain CT‐scan was reassuring with no evidence of hemorrhagic lesions, collections, or mass. Biological tests were carried out, showing a CRP of 10 mg/L, slight neutrophilic leukocytosis, a natremia of 144 mmol/L, potassium of 3.3 mmol/L, chloride of 90 mmol/L, bicarbonate of 29 mmol/L, and creatinine of 1.13 mg/dL. Liver enzymes (GOT, GPT, GGT, and PALC) were within normal limits.

The family doctor initiated betahistine treatment, without any perceived benefit.

An ENT consultation was sought. No hypoacusis, no new tinnitus, and no sensation of a blocked ear were identified. Repeated clinical examinations ruled out BPPV, and revealed fluctuating, intermittent, and multidirectional nystagmus, with cerebellar maneuvers that were sometimes well performed and sometimes dysmetric. The head impulse test was normal in both cases. The statokinetic tests were also highly variable: sometimes retropulsion, sometimes lateral deviation, and sometimes ataxia. The patient was cooperative and not confused. Severe cramping later appeared.

A neurological consultation was sought, and a cerebral MRI was ordered to rule out vascular and infectious diseases, which only identified old vasculoischemic lesions consistent with severe leukoaraiosis. After a team discussion the following day, the case of the patient described above led to the hypothesis of HiCS, which was confirmed by the presence of very low magnesium levels in the blood (< 0.24 mmol/L). It should be noted that the patient also suffered from acute renal failure in the context of dehydration linked to vomiting and increased CPK levels following cramps linked to hypomagnesemia. Biological outcome was rapidly favorable after rehydration and intravenous magnesium supplementation (3 g of MgSO4 in 1 h, then 4 g in 12 h, with normalization of magnesemia 4 h after the start of supplementation). Patient’s complaints improved significantly the following day, with the disappearance of intense dizziness. Slight instability and a slightly unsteady gait persisted.

After a thorough internal medicine assessment, which ruled out renal loss (24 h urine), the etiology of the hypomagnesemia was attributed to the PPI. The patient was discharged home with a prescription for oral magnesium (450 mg three times a day, then reduced to two times a day). Given the severity of Barrett’s esophagus, and in the absence of an effective alternative, it was decided to continue the PPI treatment. Vestibular testing, including VHIT, VNG, vestibulomyogenic evoked potentials, and vertical alignment tests, was performed up to 2.5 months after the acute episode. All results were within normal limits, with no evidence of semicircular canal or otolithic dysfunction.

Fourteen months later, the patient returned to the emergency room with similar symptoms. The patient had stopped taking oral magnesium supplements. Laboratory tests confirmed hypomagnesemia of 0.11 mmol/L. Once again, the evolution was rapidly favorable after supplementation.

## 4. Discussion

Hypomagnesemia is a rare but often overlooked cause of cerebellar syndrome. The typical clinical picture includes a subacute cerebellar syndrome (dizziness, nausea, vomiting, balance problems leading to walking difficulties, dysarthria, and ataxia) with downbeat nystagmus, accompanied by bilateral cerebellar edema of the hemispheres, vermis, or nodulus [4, 5]. Some patients may experience seizures. However, as in the second case presented, the clinical picture may fluctuate over time, complicating the differential diagnosis. Similarly, MRI may be normal despite severe clinical symptoms [4, 6, 7]. It is therefore important to evoke this diagnosis in the presence of any atypical dizziness, balance disorders, or even predominant seizures, and to measure serum magnesium, even if the imaging is normal. Patient’s observation with regular re‐examination also seems useful. A rapid response after magnesium supplementation will confirm the diagnosis, even though half of the patients may retain residual symptoms in the long term [4, 7].

Identifying the cause of the cerebellar syndrome is crucial to provide a timed, etiological treatment, as some causes may require urgent management (e.g., stroke). The differential diagnosis includes metabolic (hypoglycemia, hypocalcemia, hypomagnesemia, hyponatremia, thiamine, or vitamin E deficiency), toxic (ethanol, lithium, and heavy metals), drug‐related (antipsychotics, antiepileptics, metronidazole, and cyclosporine), infectious, immune (multiple sclerosis, systemic lupus erythematosus, Miller–Fisher syndrome, and gluten ataxia), endocrine (hypothyroidism), degenerative (idiopathic cerebellar atrophy), vascular (stroke and posterior reversible encephalopathy syndrome), neoplastic, or paraneoplastic disorders [8]. The history and overall clinical picture will often guide the diagnostic approach. Complementary exams should not be overlooked if there is a slightest doubt, including imaging to rule out vascular or oncological disorders and laboratory workup. In any case, the serum magnesium measurement is a fast, inexpensive test that enables rapid differential diagnosis, avoiding unnecessary and costly complementary exams.

Neurological disorders secondary to hypomagnesemia have been attributed to dysfunction of NMDA channels, which are particularly abundant in the cortex, hippocampus, and cerebellum [9]. Magnesium plays an inhibitory role on NMDA receptors, acting as a plug in the intracellular part of the NMDA channel, which is released when the cell membrane depolarizes. Therefore, low magnesium levels lead to lower resting potential of these neurons and subsequent hyperexcitability and increased intracellular calcium levels [10, 11]. In addition, Purkinje cells, characteristic neurons of the cerebellar cortex, are very sensitive to alterations in the level of divalent cations (calcium and magnesium); hypomagnesemia leads to hyperexcitability of these neurons by reducing the effectiveness of inhibitory neurotransmitters and increasing that of excitatory neurotransmitters (9). This could explain the predominant cerebellar dysfunction observed in severe hypomagnesemia patients.

Hypomagnesemia can arise from various mechanisms, including decreased intake, increased losses, or impaired distribution (3). It may result from insufficient dietary or enteral intake, particularly in malnourished individuals or those with chronic alcoholism. Increased gastrointestinal losses may arise in cases of chronic diarrhea, vomiting, or PPI intake. Increased renal wasting is primarily due to the use of loop and thiazide diuretics, but can also occur, for example, with aminoglycoside antibiotics or calcineurin inhibitors. In addition, a shift of magnesium from the extracellular to the intracellular compartment can contribute to decreased serum levels in certain clinical conditions, such as refeeding syndrome, acute pancreatitis, or insulin therapy, but is quite infrequent.

PPIs are the most frequently reported cause of severe hypomagnesemia and cerebellar syndrome induced by hypomagnesemia [4, 13]. The use of PPIs is associated with an increased risk of hypomagnesemia, which has been reported in up to 25% of patients [14, 15]. It would appear, however, that severe forms are relatively rare, although there are little data to quantify this. Hypomagnesemia generally develops in the months following initiation of treatment, but may also occur at a later stage, sometimes after years [16, 17]. It is therefore recommended that serum magnesium levels be monitored regularly in patients taking PPIs, and not just at the start of treatment [16]. PPI‐related hypomagnesemia is attributed to a reduction in intestinal absorption secondary to a decrease in the acidity of the intestinal fluid, which decreases the activity of magnesium absorption channels (TRPM) [18]. This is thus a class effect, with no habituation, and requires the PPI to be changed for another class of antacid, such as H2 blockers [19]. However, there is sometimes no possible alternative for some patients who are only well‐balanced on PPIs, such as the two patients presented in this paper. Discussion with the referring gastroenterologist is therefore essential. If the PPI is continued, magnesium supplementation and regular biological monitoring will be essential for as long as the PPI treatment is continued, given the rapid recurrence of the condition when the replacement treatment is stopped.

The treatment of hypomagnesemia is based on identifying the underlying cause and, if appropriate, eliminating it, as well as on magnesium supplementation. Initial supplementation depends on the severity of depletion and associated clinical symptoms [20, 21]. Mild forms (serum magnesium > 1 mEq/mL and mild or no clinical symptoms) can be corrected orally (dosage according to formulation, see package leaflet; in general, 15–20 mmol/day). More profound hypomagnesemia (< 1 mEq/mL) or symptomatic hypomagnesemia should be corrected intravenously (1–2 g/h of magnesium sulfate for the first 6 h, then 0.5–1 g/day adjusted as magnesemia progresses). When the prognosis is vital (torsade de pointe or seizures), more rapid supplementation is recommended (1–2 g of magnesium sulfate intravenously over a few minutes, followed by an infusion of 3–10 mg/min with regular biological monitoring); note that 1 g of magnesium sulfate contains 100 mg of elemental magnesium (giving 4 mmol, or 8 mEq). In the event of digestive absorption problems or intolerance to oral administration, the intravenous route is preferred. An alternative parenteral administration via the subcutaneous route has also been used successfully [22]. It should be noted, however, that despite everything, the oral route appears to be effective in the majority of cases of PPI‐induced hypomagnesemia. In cases of renal pathology, particular caution should be exercised to avoid iatrogenic hypermagnesemia (halving the dosage). Regular monitoring seems essential as nearly half of the patients have been reported to relapse [23].

## 5. Conclusion

Hypomagnesemia is a rare but overlooked cause of cerebellar syndrome. As the clinical picture can be variable, fluctuating over time, and sometimes associated with normal brain imaging, it is essential to measure magnesemia in the presence of any atypical picture of dizziness, balance disorders, or even seizures. PPI use is the most frequent cause of severe hypomagnesemia, and discounting them is not always compatible with control of digestive symptoms. As there is no habituation, supplementary magnesium treatment for as long as the PPI is taken is mandatory, with regular monitoring of magnesemia. Prompt diagnosis and treatment are essential, as some of the symptoms may not be reversible even after the magnesium has been corrected.

## Ethics Statement

This work was approved by the Ethics Committee of the CHU UCL Namur, Godinne site (nb 232/2024).

Written informed consent was obtained from both patients for publication of this case report.

## Consent

Please see the Ethics Statement.

## Conflicts of Interest

The authors declare no conflicts of interest.

## Author Contributions

Valentine Léonard: data acquisition, manuscript drafting, critical review, and final approval.

Brieuc Gevers: manuscript drafting, critical review, and final approval.

Jean‐Philippe Van Damme: critical review and final approval.

Pierre Garin: critical review and final approval.

Michaël Hardy: manuscript drafting, critical review, and final approval.

## Funding

This study was not supported by any sponsor or funder.

## Data Availability

The data that support the findings of this study are available from the corresponding author upon reasonable request.

## References

[1] Ataullah A. H. M. , Singla R. , and Naqvi I. A. , Cerebellar Dysfunction. Statpearls, 2024, Treasure Island. 32965988

[2] Jahnen-Dechent W. and Ketteler M. , Magnesium Basics, Clinical Kidney Journal. (2012) 5, no. Suppl 1, i3–i14, 10.1093/ndtplus/sfr163, 2-s2.0-84864457439. 26069819 PMC4455825

[3] Gragossian A. , Bashir K. , Bhutta B. S. , and Friede R. , Hypomagnesemia. Statpearls, 2024, Treasure Island. 29763179

[4] Kamm C. P. , Nyffeler T. , Henzen C. , and Fischli S. , Hypomagnesemia-Induced Cerebellar Syndrome-A Distinct Disease Entity? Case Report and Literature Review, Frontiers in Neurology. (2020) 11, 10.3389/fneur.2020.00968. PMC7505994 33013642

[5] Bodranghien F. , Bastian A. , Casali C. et al., Consensus Paper: Revisiting the Symptoms and Signs of Cerebellar Syndrome, The Cerebellum. (2016) 15, no. 3, 369–391, 10.1007/s12311-015-0687-3, 2-s2.0-84932167165. 26105056 PMC5565264

[6] Ray S. and Park K. W. , Movement Disorders and Other Neurologic Impairment Associated with Hypomagnesemia: a Systematic Review, Neurology Clinical Practice. (2023) 13, no. 6, 10.1212/cpj.0000000000200202. PMC10547470 37795503

[7] Wahane M. , Kaushal H. , Goyal G. , and Sarna M. , An Atypical Case of hypomagnesemia-induced Cerebellar Syndrome with Literature Review, The Journal of the Royal College of Physicians of Edinburgh. (2023) 53, no. 4, 272–277, 10.1177/14782715231209776. 37936278

[8] Manto M. and Marmolino D. , Cerebellar Ataxias, Current Opinion in Neurology. (2009) 22, no. 4, 419–429, 10.1097/wco.0b013e32832b9897, 2-s2.0-68449099404. 19421057

[9] Hansen K. B. , Yi F. , Perszyk R. E. , Menniti F. S. , and Traynelis S. F. , NMDA Receptors in the Central Nervous System, Methods in Molecular Biology. (2017) 1677, 1–80, 10.1007/978-1-4939-7321-7_1, 2-s2.0-85030760113. 28986865 PMC7325486

[10] Glitsch M. and Marty A. , Presynaptic Effects of NMDA in Cerebellar Purkinje Cells and Interneurons, Journal of Neuroscience. (1999) 19, no. 2, 511–519, 10.1523/jneurosci.19-02-00511.1999. 9880571 PMC6782199

[11] Joels M. , Yool A. J. , and Gruol D. L. , Unique Properties of non-N-methyl-D-aspartate Excitatory Responses in Cultured Purkinje Neurons, Proceedings of the National Academy of Sciences of the United States of America. (1989) 86, no. 9, 3404–3408, 10.1073/pnas.86.9.3404, 2-s2.0-0024583102. 2470102 PMC287141

[12] Darrow E. , Strahlendorf J. C. , and Strahlendorf H. K. , Extracellular Magnesium Concentration Alters Purkinje Cell Responsiveness to Serotonin and Analogues, European Journal of Pharmacology. (1991) 209, no. 1-2, 19–25, 10.1016/0014-2999(91)90005-b, 2-s2.0-0025937604. 1839984

[13] Seah S. , Tan Y. K. , Teh K. et al., Proton-Pump Inhibitor Use Amongst Patients with Severe Hypomagnesemia, Frontiers in Pharmacology. (2023) 14, 10.3389/fphar.2023.1092476. PMC9922884 36794273

[14] Park C. H. , Kim E. H. , Roh Y. H. , Kim H. Y. , and Lee S. K. , The Association Between the Use of Proton Pump Inhibitors and the Risk of Hypomagnesemia: a Systematic Review and meta-analysis, PLoS One. (2014) 9, no. 11, 10.1371/journal.pone.0112558, 2-s2.0-84911881647. PMC4230950 25394217

[15] Cheungpasitporn W. , Thongprayoon C. , Kittanamongkolchai W. et al., Proton Pump Inhibitors Linked to Hypomagnesemia: a Systematic Review and meta-analysis of Observational Studies, Renal Failure. (2015) 37, no. 7, 1237–1241, 10.3109/0886022x.2015.1057800, 2-s2.0-84941930462. 26108134

[16] Yamashiro K. , Hosomi K. , Yokoyama S. , Ogata F. , Nakamura T. , and Kawasaki N. , Adverse Event Profiles of Hypomagnesemia Caused by Proton Pump Inhibitors Using the Japanese Adverse Drug Event Report (JADER) Database, Pharmazie. (2022) 77, no. 7, 243–247, 10.1691/ph.2022.2416. 36199184

[17] Janett S. , Camozzi P. , Peeters G. G. et al., Hypomagnesemia Induced by Long-Term Treatment with Proton-Pump Inhibitors, Gastroenterology Research and Practice. (2015) 2015, 1–7, 10.1155/2015/951768, 2-s2.0-84929649776. PMC4434191 26064102

[18] Katopodis P. , Karteris E. , and Katopodis K. P. , Pathophysiology of Drug-Induced Hypomagnesaemia, Drug Safety. (2020) 43, no. 9, 867–880, 10.1007/s40264-020-00947-y. 32399868

[19] Fatuzzo P. , Portale G. , Scollo V. , Zanoli L. , and Granata A. , Proton Pump Inhibitors and Symptomatic Hypomagnesemic Hypoparathyroidism, Journal of Nephrology. (2017) 30, no. 2, 297–301, 10.1007/s40620-016-0319-0, 2-s2.0-85015734663. 27206762

[20] Ayuk J. and Gittoes N. J. , Treatment of Hypomagnesemia, American Journal of Kidney Diseases. (2014) 63, no. 4, 691–695, 10.1053/j.ajkd.2013.07.025, 2-s2.0-84897107468. 24100128

[21] Liamis G. , Hoorn E. J. , Florentin M. , and Milionis H. , An Overview of Diagnosis and Management of drug-induced Hypomagnesemia, Pharmacology Research & Perspectives. (2021) 9, no. 4, 10.1002/prp2.829. PMC8287009 34278747

[22] Tokarski R. M. , Knecht V. , Bezjak J. D. , and Ray E. C. , Home Subcutaneous Magnesium Infusion in Refractory Hypomagnesemia: a Case Report, Kidney Medicine. (2023) 5, no. 4, 10.1016/j.xkme.2023.100611. PMC10024036 36941847

[23] Olmedo-Saura G. , Perez-Perez J. , Xucla-Ferrarons T. , Collet R. , Martinez-Viguera A. , and Kulisevsky J. , Cerebellar Syndrome Induced by Hypomagnesemia: a Treatable Cause of Ataxia Not to Be Missed. Report of Three Cases and a Review of the Literature, Movement Disorders Clinical Practice. (2023) 10, no. 6. 10.1002/mdc3.13739 PMC10272920 37332648

